# Spontaneous rupture of an arachnoid cyst combined with chronic subdural hematoma: a case report and review of the literature

**DOI:** 10.3389/fsurg.2025.1671194

**Published:** 2025-10-01

**Authors:** Jinwen Zhang, Bing Han, Zengwu Wang, Fenglei Sun, Naizheng Liu

**Affiliations:** 1 School of Clinical Medicine, Shandong Second Medical University, Weifang, China; 2 Department of Neurosurgery, Weifang People’s Hospital, Shandong Second Medical University, Weifang, China

**Keywords:** arachnoid cyst, subdural hematoma, burr-hole, spontaneous, hemorrhage

## Abstract

This case report discusses a 16-year-old male with a previously diagnosed left fronto-temporo-parietal arachnoid cyst (AC) who developed progressively worsening intermittent headaches. Magnetic resonance imaging (MRI) revealed a spontaneous chronic subdural hematoma (CSDH), despite no history of trauma or bleeding disorders. The cyst, discovered incidentally three years prior, had been asymptomatic until this event; hence, no interval cranial computed tomography (CT) or MR imaging was performed. The patient underwent burr-hole irrigation and drainage under general anaesthesia. Postoperative scans confirmed complete resolution of the hematoma and a significant reduction in the cyst size, with no recurrence of the cyst at the six-month follow-up. This case, supported by a systematic review of 28 recent studies (2020–2025), highlights that AC can spontaneously rupture, leading to CSDH. Burr-hole irrigation surgery proves to be a safe and efficient intervention, emphasizing the need for long-term monitoring in AC patients to manage potential hemorrhagic complications promptly.

## Introduction

Arachnoid cysts (AC) are extra-axial cerebrospinal fluid (CSF) collections located in the arachnoid space, typically not connected to the ventricular system, and most commonly found in the middle cranial fossa. They account for approximately 1% of intracranial occupying lesions (1, 2). ACs are usually asymptomatic and most often found incidentally on cranial imaging after traumatic brain injury, making it easy to misdiagnose and miss the diagnosis (2). Chronic subdural hematoma (CSDH) is a common neurosurgical condition, most commonly seen in older adults with a history of trauma. Spontaneous CSDH accounts for approximately 3%–5% of cases, primarily due to vascular abnormalities such as aneurysms, fistulas, or arteriovenous malformations. AC is a rare cause of CSDH (3, 4). Currently, the primary surgical approaches used for symptomatic AC include neuroendoscopic fenestration, microsurgical fenestration, and cystoperitoneal shunting (5). For simple CSDH, burr hole and irrigation can often achieve significant improvement (6). However, there is some controversy about how AC contributes to the development of CSDH and the optimal surgical method for treating patients with AC-related CSDH. We retrospectively analyzed the clinical data of one case of AC with spontaneous rupture combined with CSDH admitted to Weifang People's Hospital. We discussed the clinical features, imaging manifestations, and surgical methods of AC in conjunction with literature reports to improve the diagnosis and treatment of AC.

## Case presentation

**Figure 1:**
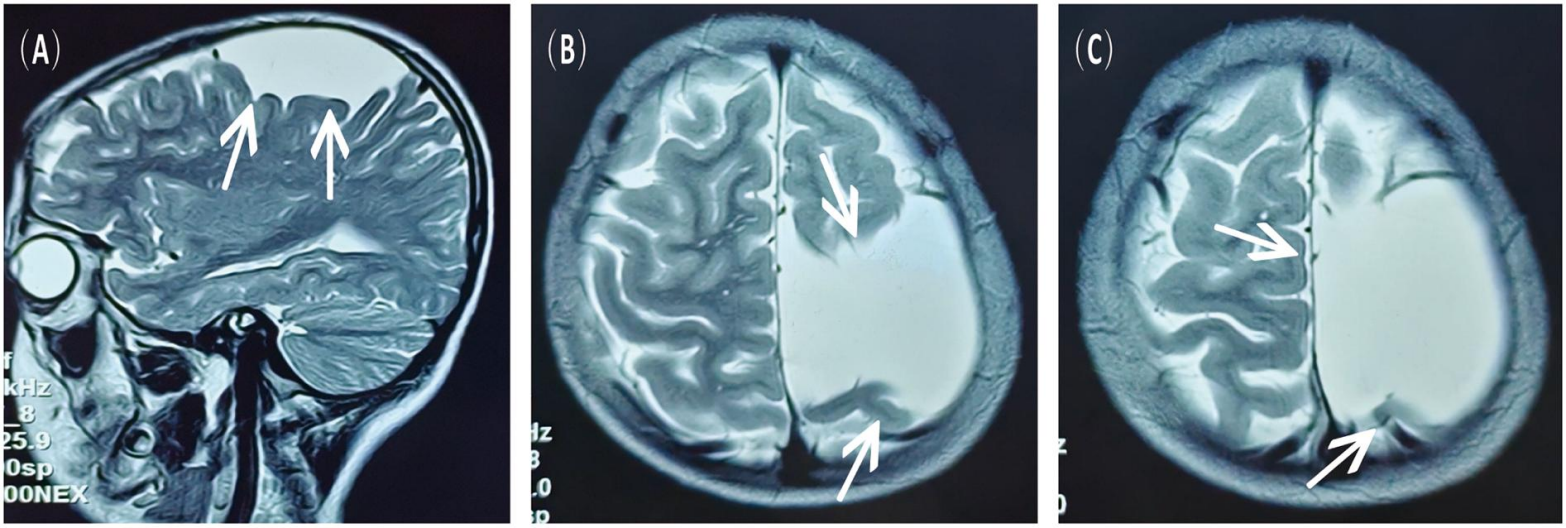
MRI scans obtained 3 years prior to the current admission, showing the left fronto-temporo-parietal arachnoid cyst. **(A)** Sagittal view, T2-weighted sequence. **(B,C)** Axial view, T2-weighted sequence.

**Figure 2:**
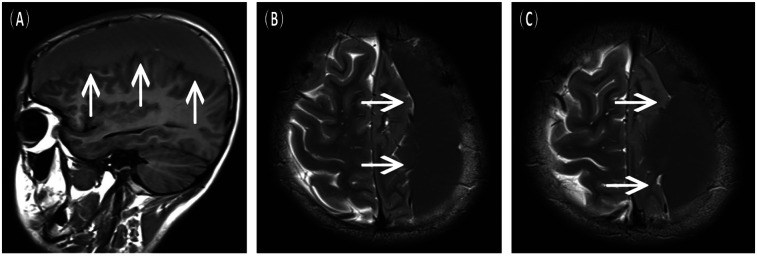
Preoperative MRI scans upon the current admission, indicative of a left chronic subdural hematoma. **(A)** Sagittal view, T1-FLAIR sequence. **(B,C)** Axial view, T2-weighted sequence.

**Figure 3:**
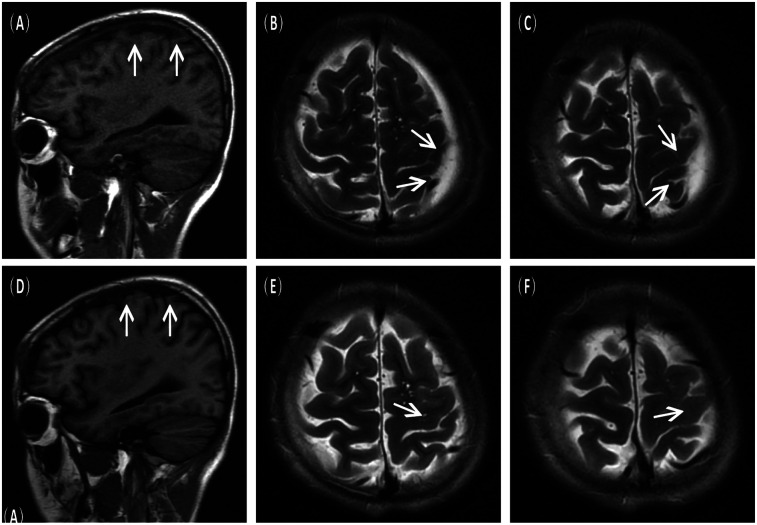
Postoperative imaging findings. **(A–C)** Immediate postoperative MRI scans showing resolution of the subdural hematoma and decreased size of the arachnoid cyst. **(D–F)** Follow–up MRI scans at 6 months post–surgery confirmed no recurrence of the cyst.

A 16-year-old male presented to the hospital with a 10-day history of episodic headache. Three years earlier, he had been evaluated for headache, and magnetic resonance imaging (MRI) identified an arachnoid cyst in the left fronto-temporo-parietal region (Figure 1). Given the absence of significant mass effect or neurological deficits and the resolution of symptoms, surgical intervention was not advised at that time. No specific intervention was performed, and the headache subsequently resolved on its own. No further imaging studies were conducted thereafter. Ten days ago, the patient experienced recurrent headaches without apparent cause or trigger, characterized by episodic attacks predominantly localized to the left occipital region. The headache was initially relieved by oral painkillers but reappeared 2 days before admission to the hospital, worsening compared to the previous episode. For further evaluation and management, the patient presented to our institution. An outpatient magnetic resonance imaging (MRI) revealed a massive chronic subdural hematoma (CSDH) in the left fronto-temporo-parietal region (Figure 2). No computed tomography angiography (CTA) or digital subtraction angiography (DSA) was performed at this time. The patient underwent burr-hole irrigation and drainage of the CSDH under general anaesthesia. Concurrently, the cyst wall was fenestrated to establish communication between the cyst and the extracerebral space. Postoperative imaging confirmed complete resolution of the hematoma and a significant reduction in the size of the cyst (Figures 3A–C). His headaches were relieved significantly compared with those of the previous day. The cyst did not recur on the patient's return visit 6 months after the operation (Figures 3D–F), and no symptoms such as headache were observed during the 6 months.

## Discussion

Arachnoid cysts (AC) are structural malformations of the arachnoid membrane that encase cerebrospinal fluid (CSF), most of which are stable and account for approximately 1% of all intracranial occupying lesions (1, 2). They are most frequently located in the middle cranial fossa and sylvian fissure, followed by the posterior cranial fossa (cerebellopontine angle, quadrigeminal cistern, and cerebellar vermis) and the suprasellar region; convexity ACs are rare (7). ACs are more common in males (1.8%) than females (1.1%) and are typically asymptomatic (2). When symptoms are present, the most common are headaches, followed by seizures, gait disturbances, and behavioral changes.

Chronic subdural hematoma (CSDH) is a common neurosurgical condition seen in older adults with a history of head trauma, and spontaneous CSDH occurs in about 3%–5% of cases, primarily due to vascular abnormalities such as aneurysms, fistulas, or arteriovenous malformations (3, 4). Robinson RG first suggested a correlation between arachnoid cysts and subdural hematomas in 1971 (8). In a study by Wu et al., the mean age of patients with AC combined with subdural hematoma was 24 years, of which 53% were under 12 years of age (9).

The exact pathogenesis of spontaneous subdural hematomas associated with AC is currently unknown. Two mechanisms have been proposed by Page et al. The first mechanism suggests that AC can amplify pressure changes in the CSF, leading to the rupture of bridging veins or vessels in the cyst wall. The second mechanism suggests that arachnoid cysts are less compliant than the normal brain, leading to reduced intracranial cushioning after minor trauma. The cystic cavity encasing the cystic fluid transfers the shear force to the outer membrane, which tears the small blood vessels between the outer arachnoid membrane and the dura mater, leading to a small amount of hemorrhage, which then progresses to the symptomatic and chronic stage of the disease (10). Hall et al. suggested that AC linings in the middle fossa may actively secrete CSF, leading to cyst expansion and eventual rupture (11). Other risk factors include bleeding diatheses, anticoagulant use, hypertension, and malignancy (12). Evidence suggests that cyst size and location are significant risk factors; middle fossa cysts, especially those extending into the sylvian fissure, are more prone to rupture due to proximity to vascular structures and temporal lobe dynamics (13, 14). In terms of imaging, magnetic resonance imaging (MRI) is the primary diagnostic tool for intracranial arachnoid cyst screening, which can help identify cysts not only with other protein-rich lesions (e.g., epidermoid cysts), but also with various cystic pathologies (e.g., tumors or infections) (2). According to Osborn et al., differentiation between arachnoid cysts and chronic or subacute subdural hematomas or intracystic hemorrhages can be challenging when proteinaceous or hemorrhagic contents alter the cystic fluid signal intensity on MRI (15). In this case, a massive hemorrhage interfered significantly with the imaging of AC. Additionally, computed tomography angiography (CTA) and digital subtraction angiography (DSA) are increasingly recommended to identify underlying vascular pathologies, such as dural arteriovenous fistulae, which may contribute to hematoma formation or recurrence, particularly in cases of spontaneous or recurrent CSDH (16). This diagnostic approach is especially relevant in AC-associated CSDH, where altered intracranial dynamics and mechanical stress on adjacent vessels may predispose to vascular abnormalities.

A meta-analysis by Chen et al. of 474 patients with middle fossa ACs compared neuroendoscopic fenestration, microsurgical fenestration, and cystoperitoneal shunting. Neuroendoscopic fenestration was safest, though all techniques were equally effective (5). For CSDH, burr-hole irrigation remains the standard treatment (6). Of the published cases of CSDH associated with AC, some were treated conservatively, but the vast majority were treated surgically. There is some controversy about the optimal surgical approach, craniotomy or burr hole and irrigation, for surgery for AC combined with CSDH (9, 15). We selected burr-hole irrigation and drainage based on several considerations. First, this technique effectively evacuates the hematoma, eliminates inflammatory and pro-angiogenic mediators (e.g., VEGF and IL-6), and disrupts the cycle of recurrent bleeding and exudation, a pathophysiological hallmark of CSDH (17). Second, fenestration of the cyst wall during the same procedure facilitates communication between the cyst and the subarachnoid space, promoting cyst collapse and reducing the risk of recurrence (5, 9). Although craniotomy with cyst excision offers direct visualization, it is associated with greater tissue injury, longer operative time, and higher morbidity, particularly in young patients.

In contrast, burr-hole irrigation surgery is minimally invasive, cost-effective, and demonstrates excellent clinical outcomes with low recurrence rates, as evidenced by our case and multiple series (6, 18). Sayer reviewed 79 patients with AC-related CSDH published from 1994 to 2019, of which 71 were successfully treated by burr hole and irrigation, and none of them required repeat craniotomy at long-term follow-up (13). To ensure a comprehensive and updated overview of AC-associated chronic subdural hematoma (CSDH), we conducted a systematic literature search on PubMed and Google Scholar for case reports published between 2020 and 2025 (Table 1). The keywords used included arachnoid cyst, subdural hematoma, chronic subdural hematoma, and spontaneous hemorrhage. Only reports describing subacute or chronic subdural hematomas associated with arachnoid cysts, with clearly documented management and outcomes, were included.

**Table 1:** Summary of reported cases of arachnoid cyst-associated subdural hematoma (2020–2025).

#	Author/year	Sex/age, years	Cyst location	Etiology	Treatment	Outcome
1	Gregori/2020 (22)	M/10	Middle fossa	Trauma-related	Burr-hole irrigation	Improved
2	Gregori/2020 (22)	M/18	Middle fossa	Spontaneous	Burr-hole irrigation	Improved
3	Gregori/2020 (22)	M/6	Middle fossa	Trauma-related	Craniotomy + fenestration	Improved
4	Chung/2020 (23)	M/3	Middle fossa	Spontaneous	Craniotomy + fenestration	Improved
5	Balestrino/2020 (13)	M/7	Posterior fossa	Trauma-related	Craniotomy + fenestration	Improved
6	Balestrino/2020 (13)	M/10	Middle fossa	Spontaneous	Burr-hole irrigation	Improved
7	Balestrino/2020 (13)	M/5	Middle fossa	Spontaneous	Burr-hole irrigation	Improved
8	Balestrino/2020 (13)	F/13	Middle fossa	Trauma-related	Craniotomy + fenestration	Improved
9	Balestrino/2020 (13)	M/0.25	Middle fossa	Spontaneous	Burr-hole irrigation	Improved
10	Sutiono/2020 (24)	M/18	Middle fossa	Trauma-related	Craniotomy + fenestration	Improved
11	Kieu/2021 (25)	F/33	Middle fossa	Spontaneous	Craniotomy + fenestration	Improved
12	Sayer/2022 (18)	M/14	Middle fossa	Spontaneous	Burr-hole irrigation	Improved
13	Shimizu/2022 (26)	M/16	Convexity	Spontaneous	Burr-hole irrigation	Improved
14	Karakoc/2023 (27)	M/16	Sylvian fissure	Spontaneous	Burr-hole irrigation	Improved
15	Hanai/2023 (28)	M/18	Convexity	Spontaneous	Conservative	Improved
16	Kim/2023 (29)	M/12	Middle fossa	Trauma-related	Burr-hole irrigation	Improved
17	Kim/2023 (29)	M/14	Middle fossa	Spontaneous	Craniotomy + fenestration	Improved
18	Kim/2023 (29)	M/17	Posterior fossa	Spontaneous	Burr-hole irrigation	Improved
19	Borni/2023 (30)	M/16	Middle fossa	Trauma-related	Conservative	Lost to FU
20	Nguyen/2023 (14)	M/18	Middle fossa	Spontaneous	Craniotomy + fenestration	Improved
21	Nguyen/2023 (14)	M/14	Middle fossa	Spontaneous	Craniotomy + fenestration	Improved
22	Nguyen/2023 (14)	M/21	Middle fossa	Spontaneous	Burr-hole irrigation	Improved
23	Lines-Aguilar/2024 (4)	M/23	Middle fossa	Spontaneous	Craniotomy + fenestration	Improved
24	Cedeño-Morán/2024 (31)	M/10	Middle fossa	Spontaneous	Burr-hole irrigation	Improved
25	Elkazaz/2024 (32)	F/19	Middle fossa	Spontaneous	Craniotomy + fenestration	Improved
26	Asagiri/2024 (33)	M/27	Sylvian fissure	Spontaneous	Craniotomy + fenestration	Improved
27	Bilgin/2024 (34)	F/35	Middle fossa	Spontaneous	Conservative	Stabilized
28	This report	M/16	Fronto-temporo-parietal	Spontaneous	Burr-hole irrigation	Improved

M, male; F, female; FU, follow-up.

A total of 28 cases were identified and analyzed with respect to patient demographics, etiology, treatment modality, and clinical outcomes. The majority of patients were male (*n* = 24, 85.7%), with a mean age of 15.3 years (range: 3 months–35 years)—most cases (*n* = 21,75%) involved middle cranial fossa cysts. A history of minor or unrecognized trauma was reported in 7 cases (25.0%), while the remainder were truly spontaneous.

Treatment approaches were categorized as follows: burr-hole irrigation (*n* = 13, 46.4%), craniotomy with cyst fenestration or excision (*n* = 12, 42.9%), and conservative management (*n* = 3, 10.7%). All surgically treated patients showed significant clinical improvement—notably, none of the patients treated with burr-hole irrigation experienced recurrence during follow-up. Among conservatively managed cases, one patient was lost to follow-up, one showed hematoma stability, and one improved. These findings align with earlier systematic reviews (9) confirming the efficacy and safety of burr-hole evacuation, particularly in cases without significant membranous organization or solid components. This review confirms burr-hole irrigation as an effective first-line treatment for AC-related CSDH.

In recent years, middle meningeal artery (MMA) embolization has emerged as a minimally invasive therapeutic option for refractory or recurrent CSDH, targeting the pathological neovascularization and inflammatory cascade that perpetuates hematoma persistence (19, 20). Although our patient was successfully managed with burr-hole irrigation and cyst fenestration without the need for additional vascular intervention, the evolving literature suggests that preoperative vascular imaging could be considered in cases of spontaneous CSDH, particularly in younger patients without clear risk factors. Future studies are warranted to define the role of CTA/DSA and prophylactic MMA embolization in the specific context of AC-associated CSDH, which may further optimize treatment strategies and reduce recurrence risks (21).

## Conclusion

In summary, AC may rarely cause spontaneous subdural hemorrhage without significant trauma. Adolescents and young adults with acute symptoms such as persistent headache should be evaluated for cyst-related complications like CSDH, even without a traumatic history. Long-term monitoring is advised for known AC, especially those in the middle fossa or of larger size. Burr-hole irrigation and drainage, combined with cyst fenestration, represents a safe, effective, and minimally invasive treatment that addresses both the hematoma and the underlying pathological mechanism, thereby reducing recurrence risk. This approach provides satisfactory clinical outcomes and should be considered as a first-line surgical option for similar cases.

## Data Availability

The original contributions presented in the study are included in the article/Supplementary Material, further inquiries can be directed to the corresponding author.
